# I-waves in motor cortex revisited

**DOI:** 10.1007/s00221-020-05764-4

**Published:** 2020-03-17

**Authors:** Ulf Ziemann

**Affiliations:** 1 Department of Neurology and Stroke, University of Tübingen, Hoppe-Seyler-Str. 3, 72076 Tübingen, Germany; 2 Hertie Institute for Clinical Brain Research, University of Tübingen, Tübingen, Germany

**Keywords:** I-waves, Motor cortex stimulation, Transcranial magnetic stimulation, Epidural spinal cord potential recording, Motor cortical interneuronal circuits, Neuronal oscillator

## Abstract

I-waves represent high-frequency (~ 600 Hz) repetitive discharge of corticospinal fibers elicited by single-pulse stimulation of motor cortex. First detected and examined in animal preparations, this multiple discharge can also be recorded in humans from the corticospinal tract with epidural spinal electrodes. The exact underpinning neurophysiology of I-waves is still unclear, but there is converging evidence that they originate at the cortical level through synaptic input from specific excitatory interneuronal circuitries onto corticomotoneuronal cells, controlled by GABAAergic interneurons. In contrast, there is at present no supportive evidence for the alternative hypothesis that I-waves are generated by high-frequency oscillations of the membrane potential of corticomotoneuronal cells upon initial strong depolarization. Understanding I-wave physiology is essential for understanding how TMS activates the motor cortex.

## Introduction

**Fig. 1:**
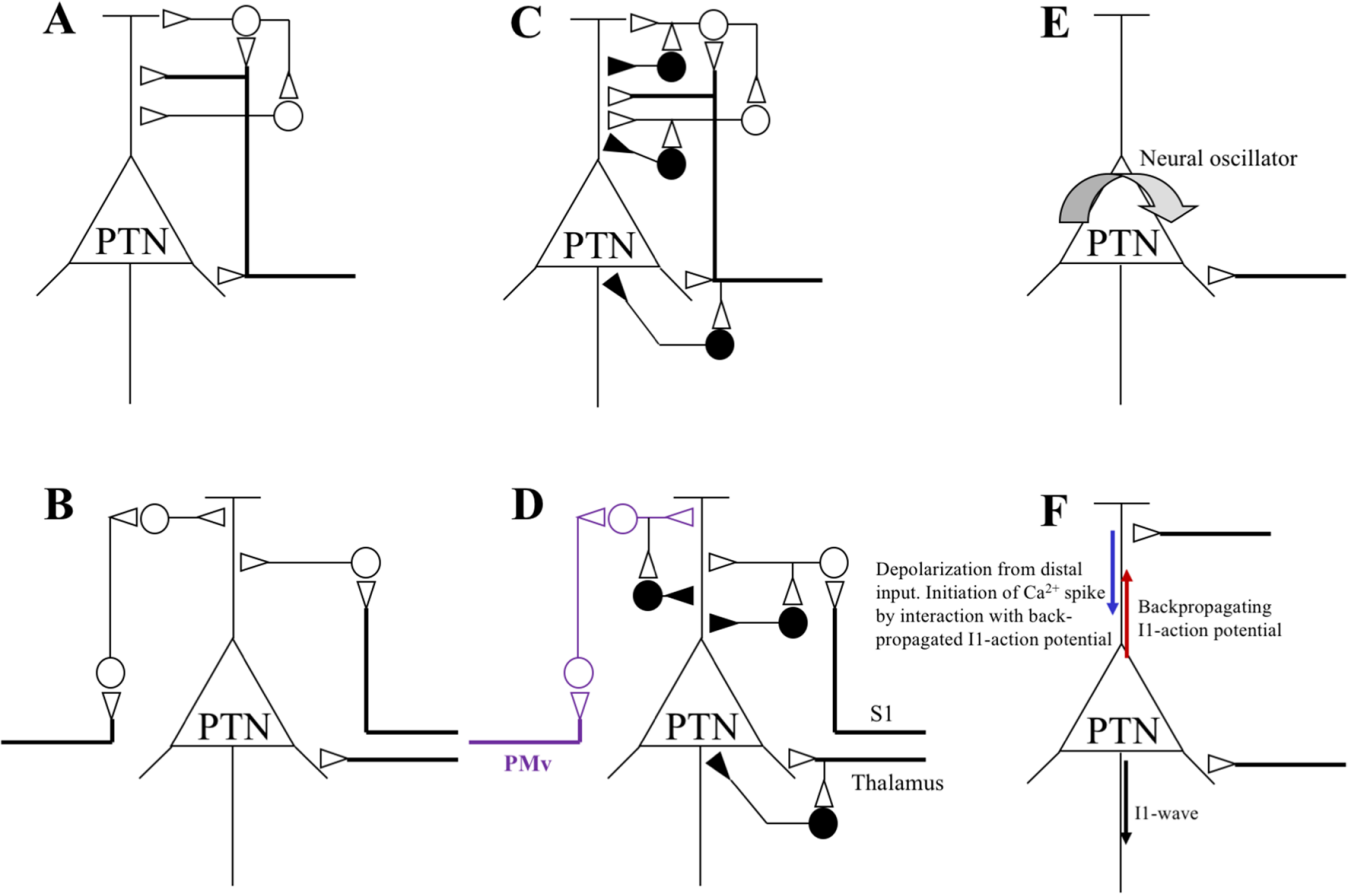
Hypothetical models (**a**–**e**) for I-wave generation. The triangular neuron is a pyramidal tract neuron (corticomotoneuronal cell, PTN). Open circles denote excitatory interneurons, while filled circles are inhibitory ones. Their synapses onto the PTN are shown by small triangles. Thick lines refer to axons which are thought to be excited by transcranial magnetic stimulation. Model **a** is similar to the one developed by Patton and Amassian (Patton and Amassian 1960; Amassian et al. 1987). It assumes periodic bombardment of PTNs through chains of interneurons with fixed temporal characteristics. Model **b** is a variation of model **a**: repetitive I-wave discharge is produced by activation of independent chains of interneurons, each responsible for generating a different I-wave (Day et al. 1989; Sakai et al. 1997; Di Lazzaro et al. 2001). Models **c** and **d** are identical to models **a** and **b**, but implement GABAAergic inhibitory interneurons that control I-wave generation along the excitatory interneuron pathways (Di Lazzaro et al. 2000; Shimazu et al. 2004). Model **d** also indicates possible sources of I-wave pathways projecting to PTNs in primary motor cortex (PMv, ventral premotor cortex; S1, primary somatosensory cortex). Conclusive causal evidence has been provided so far for the I-wave pathway from PMv only (indicated by purple color). Model **e** assumes that surface stimulation of the motor cortex produces strong and synchronized depolarization of many corticospinal cells (or interneurons), which leads to oscillatory activity and repetitive discharge of these cells as a product of their intrinsic membrane properties (Creutzfeldt et al. 1964; Phillips 1987). Model **f** proposes that repetitive firing of the PTN results from backpropagation of an action potential generated at the initial axon segment into the apical dendrite where it produces a calcium action potential upon integration with additional synaptic depolarization (Larkum et al. 1999, 2001; Ugawa et al. 2019). This figure is adopted from Fig. 3 in (Ziemann and Rothwell 2000), with permission

The initial part of this text is based on an earlier publication (Ziemann and Rothwell 2000). This paper had already concluded from the evidence that was available 20 years ago that I-waves are generated, most likely, at the cortical level through synaptic input from specific excitatory interneuronal circuitries onto corticomotoneuronal cells, controlled by GABAAergic interneurons. Several other I-wave models were discussed and discarded. These models are summarized in Fig. 1 (adopted from Fig. 3 in Ziemann and Rothwell (2000)). Revisiting here the I-wave models implies that the previous evidence, and the evidence added since then will be scrutinized for its falsifying of verifying/supporting value of these I-wave models.

## Phenomenology and terminology

Adrian and Moruzzi were the first to reveal details of the physiology of sensorimotor cortex stimulation by recording responses directly from single axons or small groups of fibers of the corticospinal tract in cats (Adrian and Moruzzi 1939). They demonstrated that the descending pyramidal discharge can take the form of high-frequency bursts of up to 500–1000 Hz, in particular when convulsant drugs such as strychnine or picrotoxin were applied to the motor cortex (M1). Patton and Amassian (1954) examined, in a highly influential study, how single-pulse electrical stimulation of the exposed M1 of cats and monkeys could give rise to multiple descending volleys in the corticospinal tract at a discharge rate of ~ 600 Hz (Patton and Amassian 1954). They provided evidence that the initial volley was caused by direct excitation of the corticospinal axons, while all later volleys were due to indirect, synaptic activation of the corticospinal neurons. Accordingly, they coined the terms D- (direct) and I- (indirect) waves to describe these responses (Patton and Amassian 1954, 1960). While the D-wave persisted during anesthesia or after cortical ablation, I-waves were abolished, indicating that they require intact and excitable gray matter (Patton and Amassian 1954, 1960). Also, local injection of the GABAA receptor agonist muscimol into M1 resulted in abolition of late I-waves, strong reduction of the I1-wave, but no effect on the D-wave (Shimazu et al. 2004). Following the inauguration of transcranial electrical stimulation (TES) (Merton and Morton 1980) and transcranial magnetic stimulation (TMS) (Barker et al. 1985), similar multiple descending discharges were observed in epidural recordings from the human spinal cord in patients undergoing spinal or brain surgery (Boyd et al. 1986; Inghilleri et al. 1989; Berardelli et al. 1990; Burke et al. 1990, 1992, 1993; Hicks et al. 1992; Rothwell et al. 1994; Fujiki et al. 1996, 2006; Kaneko et al. 1996a), and even in conscious non-anesthetized patients with implanted electrodes into the spinal epidural space for control of otherwise intractable pain (Kaneko et al. 1996b; Nakamura et al. 1996, 1997; Di Lazzaro et al. 1998a, b, 2013) (for review, Di Lazzaro and Ziemann (2013)). However, epidural spinal cord recordings are invasive and only rarely available. The physiology of D- and I-waves can be tested alternatively by single motor unit recordings using needle electromyography (EMG) (Day et al. 1987, 1989; Boniface et al. 1991; Mills 1991; Awiszus and Feistner 1994a, b; Ziemann et al. 2004). These studies provide information about the synaptic input to single spinal motoneurons and have demonstrated that they receive a sequence of excitatory postsynaptic potentials (EPSPs) consistent with arrival of multiple monosynaptic corticomotoneuronal inputs from D- and I-waves. One important limitation is that the responses recorded from single motoneurons in the needle EMG are contaminated by other inputs from activation of spinal circuitry by the corticospinal volley. For example, Ia inhibitory interneurons are also activated, which then project onto motoneurons. The consequence is that corticospinal activity can result in a sequence of EPSP/inhibitory postsynaptic potentials (IPSPs) at the motoneuron (see Cowan et al. 1986).

## Site of generation of I-waves

A variety of animal experiments have been conducted to determine which neural elements are responsible for generating the excitatory input to pyramidal neurons upon electrical M1 stimulation (Amassian et al. 1987).

One candidate are thalamocortical projections from the lateral and anterior ventral thalamic nuclei, which have monosynaptic excitatory access large pyramidal tract neurons and excitatory interneurons in the cat (Amassian and Weiner 1966). However, massive lesions of the thalamus and thalamocortical afferents did not typically have significant impact on I-wave generation (Amassian et al. 1987). Therefore, projections from anterior and lateral ventral thalamus to M1 are not essential for the production of I-waves.

M1 also receives afferent excitatory input from surrounding cortex, in particular from ventral and dorsal premotor cortex, supplementary motor area, and somatosensory cortex via long-range cortico-cortical fibers (Matsumara and Kubota 1979; Muakassa and Strick 1979; Jones 1983; DeFelipe et al. 1986; Dum and Strick 2005). Surface stimulation of these areas resulted in large repetitive I-waves in the pyramidal tract, which were abolished after ablation of M1 (Patton and Amassian 1960), suggesting that I-waves can originate by activation of cortico-cortical input to corticomotoneuronal cells. Removal of precentral cortex abolished the I-waves, indicating that they can be mediated by synaptic activation of corticomotoneuronal cells via input from premotor cortex (Amassian et al. 1987). Similar lesion or cooling experiments of somatosensory (postcentral) cortex have not been conducted. Electrophysiological experiments in monkeys demonstrated that conditioning stimulation of ventral premotor cortex facilitated the I2- and I3-waves but not the D- or I1-wave elicited by M1 stimulation, at interstimulus intervals < 1 ms (Shimazu et al. 2004). This facilitatory interaction was inhibited by local M1 injection of the GABAA receptor agonist muscimol (Shimazu et al. 2004). These findings indicate that cortico-cortical inputs from ventral premotor cortex to M1 impinge on excitatory interneurons generating late I-waves, controlled by local inhibitory interneurons.

In summary, these experiments in cat and monkey provide evidence that I-waves are generated synaptically through activation of cortico-cortical fibers impinging on excitatory interneurons in M1, or projecting to M1, that give rise to specific I-waves.

In humans, TMS activates the M1 hand area at a depth of 1.5–2.1 cm (Epstein et al. 1990), which is at the level of the deep cortical layers or at the gray–white matter border. I-waves are elicited best, if the induced current in the brain is directed from lateral-posterior to medial-anterior, approximately perpendicular to the central sulcus, while D-waves are produced preferentially if the current runs from lateral to medial, i.e., in parallel to the central sulcus (Mills et al. 1992; Werhahn et al. 1994; Kaneko et al. 1996a; Sakai et al. 1997) (for review, Di Lazzaro et al. (2004)). TMS activates fibers at lowest threshold if they run for some distance in parallel with the induced electrical field (Amassian et al. 1992; Laakso et al. 2018). This orientation selectivity is in agreement with activation of cortico-cortical fibers from premotor and/or somatosensory cortex, which predominantly run along the anterior/posterior axis.

Paired-pulse TMS of M1 has provided circumstantial information about the nature of the neural elements responsible for I-wave generation (Amassian et al. 1996; Tokimura et al. 1996; Ziemann et al. 1998a; Ziemann et al. 1998b; Di Lazzaro et al. 1999b; Rothwell 1999; Hanajima et al. 2002; Ilic et al. 2002; Wagle-Shukla et al. 2009; Delvendahl et al. 2014; Van den Bos et al. 2018b). Short-interval intracortical facilitation (SICF) occurs at specific interstimulus intervals of 1.1–1.5 ms, 2.3–2.9 ms and 4.1–4.4 ms, and if the intensity of both pulses is either around motor threshold (Tokimura et al. 1996) or if a suprathreshold first pulse and a subthreshold second pulse are applied (Ziemann et al. 1998a). There is no facilitation at other timings. The intervals of ~ 1.5 ms between the facilitatory peaks closely matches the latencies between successive I-waves in epidural spinal cord recordings (see above). Therefore, by analogy, it was suggested that SICF reflects facilitatory I-wave interaction (Tokimura et al. 1996; Ziemann et al. 1998a). The intracortical origin of SICF was conclusively demonstrated by epidural spinal cord recordings that showed larger and more numerous I-waves with paired-pulse TMS at short interstimulus intervals of 1.0–1.4 ms than expected from the arithmetic sum of each stimulus alone (Di Lazzaro et al. 1999b).

**Fig. 2:**
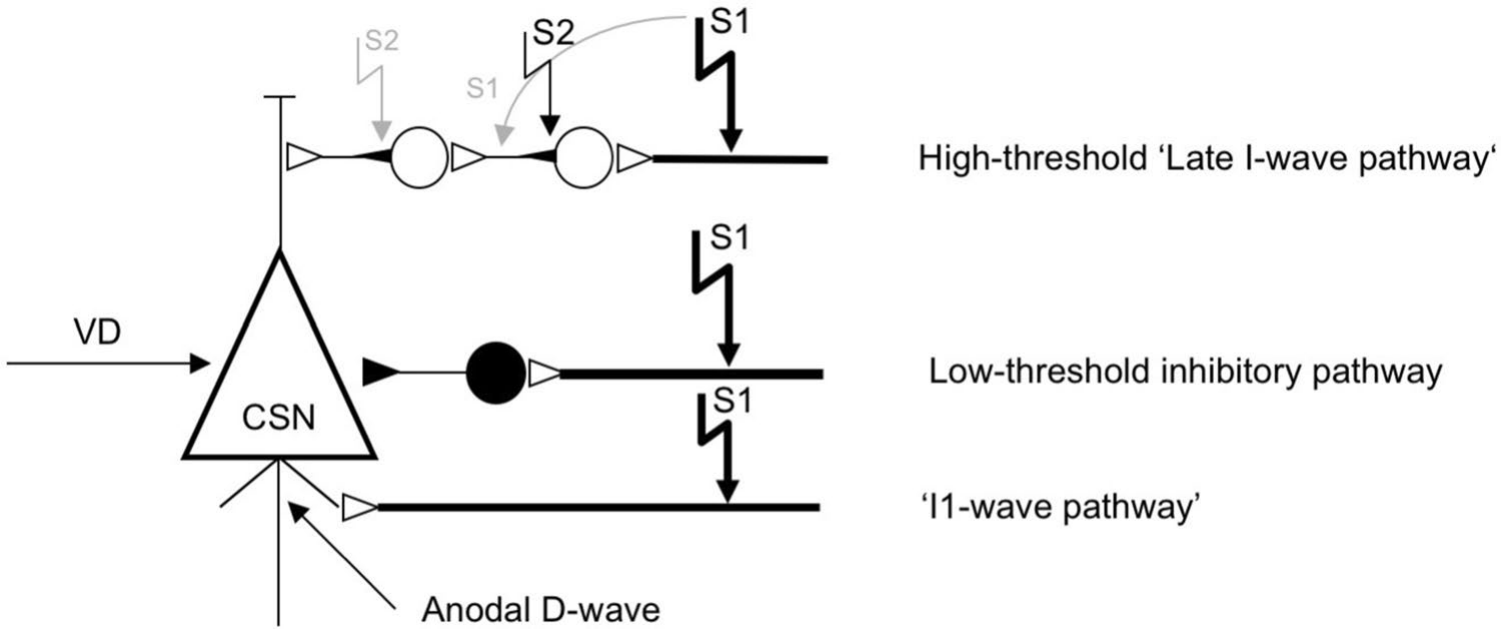
The connectivity model is derived from Fig. 4 in (Amassian et al. 1987). The model is a gross simplification but it is sufficient to explain all experimental data. It assumes that there exists one low-threshold inhibitory pathway, and high-threshold excitatory ‘I1- and late I-wave pathways’. CSN, corticospinal neuron; VD, voluntary drive. Closed circle denotes a GABAAergic inhibitory interneuron, open circle are excitatory interneurons. To explain short-interval intracortical facilitation (SICF) as tested by paired-pulse transcranial magnetic stimulation, a high-intensity first stimulus (S1) and a low-intensity second stimulus (S2) are applied. S1 activates all pathways. S2 cannot activate any axon due to refractoriness. However, the initial axon segment of the second-order interneuron in the ‘late I-wave pathway’ (indicated by the small filled triangle adjacent to the cell soma) is hyperexcitable due to the excitatory postsynaptic potential (EPSP) from S1 and can be excited directly by S2. Therefore, the site of excitation by S2 ‘jumps up’ by one I-wave latency, and the facilitatory interaction between S1 and S2 lags the anodal D-wave latency by only two I-wave intervals. In some instances, S1 may activate in addition the axon of some second-order interneurons (indicated by the gray curved arrow). In this case, the initial axon segment of first-order interneurons is hyperexcitable due to the EPSP from S1 and can be excited by S2. The facilitatory interaction between S1 and S2 would then lag the anodal D-wave latency by only one I-wave interval (from Fig. 7B in (Ilic et al. 2002), with permission)

Which neural elements are excited by the subthreshold or close to motor threshold second stimulus? A pair of anteriorly directed cathodal electrical stimuli did not produce MEP facilitation at an interstimulus interval of 1.2 ms (Amassian et al. 1998), suggesting that cortico-cortical fibers are refractory at such short intervals. Single motor unit recordings demonstrated that SICF occurs at the I2- and sometimes even the I1-wave latency of the second stimulus (Hanajima et al. 2002; Ilic et al. 2002). One parsimonious explanation for its occurrence at discrete interstimulus intervals is that the second stimulus directly excites the initial axon segment of those excitatory interneurons, which had received an EPSP from the first stimulus but have not fired an action potential and, therefore, are hyperexcitable at the time of second stimulus (Fig. 2) (Amassian et al. 1990; Deletis et al. 2001; Ilic et al. 2002). If the second stimulus is weaker than the first stimulus, then there is virtually no other explanation, due to refractoriness of those neural elements excited by the first stimulus. The neuronal time constant of the initial axon segment is probably very short. Interneurons in rat visual cortex have chronaxies of ~ 300 µs (Nowak and Bullier 1998). If this were true for the initial axon segments of interneurons in the late I-wave pathway (Fig. 2), then shifting the interstimulus interval between the first and second stimulus away from the I-wave interval (i.e., ~ 1.5 ms) should result in rapid decay or even lack of SICF. The second stimulus would then hit initial axon segments that are not yet or no longer hyperexcitable. In summary, SICF originates non-synaptically through direct excitation of the axon initial segment of excitatory interneurons of the late I-wave pathway by the second stimulus, which were made hyperexcitable through EPSPs by the first stimulus. This way, SICF is not equivalent to I-waves, but acts upon a chain of excitatory interneurons that mediate I-waves.

## Physiology of I-waves

The previous paragraphs have summarized the evidence that I-waves are produced in M1 through activation of neural elements presynaptic to corticomotoneuronal cells. But how exactly this happens is still not fully clear. In a previous review on this topic (Ziemann and Rothwell 2000), five I-wave models (models A–E) were discussed (Fig. 1).

Model A explains very elegantly the regular and rhythmic nature of the repetitive I-wave discharge. Recordings from single corticospinal axons showed that they can discharge at I-wave frequency (~ 600 Hz) (Patton and Amassian 1954; Kernell and Chien-Ping 1967), consistent with bombardment by repetitive excitatory input. However, model A cannot explain several experimental observations: (1) different I-waves are sensitive to different orientations of the induced current so that they even can be elicited in isolation (Day et al. 1989; Sakai et al. 1997). In active hand muscles, single motor unit recordings showed that the I1-wave is produced preferentially by currents in posterior-to-anterior (PA) direction, while the I3-wave is elicited preferentially by currents in the opposite, i.e., anterior-to-posterior (AP) direction (Day et al. 1989; Sakai et al. 1997); (2) this was confirmed by I-wave recordings from the epidural spinal space (Di Lazzaro et al. 2001). Furthermore, I-waves evoked by AP stimulation often had slightly different peak latencies and/or longer duration than those evoked by PA stimulation, and the relationship between the size of the I-waves and the motor evoked potential (MEP) amplitude was often different for AP and PA stimulation (Di Lazzaro et al. 2001). These findings strongly suggest that AP stimulation does not simply activate a subset of the sites activated by PA stimulation. Some sites or axons that are relatively inaccessible to PA stimulation may be the low-threshold targets of AP stimulation, and vice versa; (3) SICF experiments with paired-pulse TMS, using slightly suprathreshold intensities of 105% MEP threshold for both the first and second TMS pulse, demonstrated that monophasic AP–AP stimulation resulted in stronger early facilitation at an interstimulus interval of 1.4 ms relative to longer intervals of 2.8 and 4.4 ms, whereas monophasic PA–PA stimulation produced SICF of comparable size at all three intervals (Delvendahl et al. 2014). The conclusion is that I-waves cannot be mediated through one pathway of excitatory interneurons with fixed temporal characteristics, but some I-waves are generated through different chains of cortical excitatory interneurons than other I-waves. Model B would fit these results (Fig. 1).

Model B, but not model A, would also explain another important observation: the selective modification of some I-waves but not others by experimental manipulation. Single motor unit recordings, as well as direct epidural recordings of descending corticospinal volleys from spinal cord showed that late I-waves, particularly the I3- and later I-waves are significantly inhibited by a subthreshold TMS stimulus given through the same coil prior to the test stimulus at short (2–5 ms) (Nakamura et al. 1997; Di Lazzaro et al. 1998c; Hanajima et al. 1998) or long (100–150 ms) interstimulus intervals (Di Lazzaro et al. 2002b), a conditioning TMS pulse applied to M1 of the opposite hemisphere (Di Lazzaro et al. 1999a) or short-latency inhibition produced by electrical stimulation of the median nerve at the wrist of the contralateral hand (Tokimura et al. 2000), while the I1-wave remained unaffected. Similarly, repetitive TMS (rTMS) for induction of long-term change of corticospinal excitability resulted predominantly in modulation of the late I-waves: Low-frequency (1 Hz) regular rTMS (Di Lazzaro et al. 2008b) and paired-associative stimulation at a short interstimulus interval (10 ms) (Di Lazzaro et al. 2009b) resulted in depression of MEP amplitude and late I-waves, but not the I1-wave. In contrast, suprathreshold high-frequency (5 Hz) regular rTMS (Di Lazzaro et al. 2002a), intermittent theta-burst stimulation (Di Lazzaro et al. 2008a) and paired-associative stimulation (interstimulus interval, 25 ms) (Di Lazzaro et al. 2009a) led to increase of MEP amplitude and increase in the amplitude and/or number of late I-waves, but not the I1-wave. Even more importantly, continuous theta-burst stimulation resulted in depression of MEP amplitude and selective decrease of the I1-wave, while all late I-waves remained unaffected (Di Lazzaro et al. 2005). The conclusion must be that the I1-wave is produced by a different anatomical substrate and mechanism than the late I-waves.

However, models A and B (Fig. 1) do not explain the powerful GABAAergic inhibitory control of I-waves as demonstrated in neuropharmacological experiments. Volatile and intravenous anesthetics enhance neurotransmission through the GABAA receptor and lead to marked depression of I-waves in epidural spinal cord recordings (Hicks et al. 1992; Burke et al. 1993; Kitagawa et al. 1995; Woodforth et al. 1999). In paired-pulse TMS experiments, benzodiazepines and barbiturates, i.e., positive allosteric modulators as the GABAA receptor, inhibited SICF (Ziemann et al. 1998b; Ilic et al. 2002), while baclofen, a specific agonist of the GABAB receptor and glutamatergic *N*-methyl-d-aspartate receptor antagonists had no effect (Ziemann et al. 1998b) (for review, (Ziemann et al. 2015). Also, carbamazepine, a voltage-gated sodium channel blocker, had no effect, if the intensity of the second stimulus was adjusted to compensate for the increase in motor threshold (Ziemann et al. 1998b). Moreover, triple-pulse TMS experiments showed that SICF is reduced in the presence of GABAAergic short-interval intracortical inhibition (Shirota et al. 2010), while it is enhanced during late cortical disinhibition (Cash et al. 2011). Models C and D in Fig. 1 are variations of models A and B that account for this broad evidence of GABAAergic inhibitory control of I-waves by the insertion of inhibitory interneurons. Single-nucleotide polymorphisms of the transient receptor potential vanilloid 1 (TRPV1) channels increase presynaptic release of glutamate and these polymorphisms were associated with a selective increase in SICF peaks (Mori et al. 2012). These results are directly compatible with the view that glutamate is the neurotransmitter in the proposed chains of excitatory interneurons responsible for the generation of I-waves. Similarly, in addition to its inhibitory effect on monoamine oxidase-type B, the anti-parkinsonian drug safinamide inhibits presynaptic glutamate release through blockage of voltage-gated sodium channels and results in significant suppression of SICF in patients with Parkinson’s disease and levodopa-induced dyskinesias (Guerra et al. 2019).

Another, very different model to explain I-wave periodicity is to conceive corticomotoneuronal cells as neural oscillators (Creutzfeldt et al. 1964; Phillips 1987) (model E in Fig. 1). If their membrane properties were appropriate, a single stimulus could cause long-lasting depolarization and lead to repetitive discharge. This model predicts that the second stimulus of paired-pulse TMS will produce facilitation only if its input arrives during an epoch of increased firing probability following the first stimulus. However, the intrinsic membrane properties of corticospinal cells are unknown yet. Some results even point against fast oscillations of corticospinal cells. Large layer V pyramidal cells of cat M1 (which however were not verified as corticospinal cells) showed very narrow spikes, but a shallow firing rate-to-intensity slope (Chen et al. 1996). The short duration of their action potentials suggests that these cells are capable of firing at very high rates. However, injection of depolarizing currents was not sufficient to drive these cells to fast rates (Chen et al. 1996). Instead, this may require repeated EPSPs to arrive in close succession at the cell soma. Moreover, anodal direct current stimulation resulted in a significant increase in the D-wave, I1-wave and late I-waves, indicating a non-synaptic polarizing mechanism (Di Lazzaro et al. 2013). Remarkably, the enhancing effect on the D-wave outlasted the effect on the I-waves, which is incompatible with the idea of a neural oscillator that would predict a parallel time course of change of all waves. A recently suggested model has specified the hypothetical neural oscillator (model E in Fig. 1) by proposing that initial perisomatic monosynaptic excitation of corticomotoneuronal cells discharges the cell at the initial axon segment and evokes an I1-wave. This I1-activity backpropagates to the apical dendrite where it integrates with additional synaptic depolarization to produce a calcium action potential that is sufficiently large to produce a second action potential (and possibly a third or even more action potentials, depending on the strength of dendritic depolarization) at the initial axon segment. The interval between I1- and I2-waves relates to the conduction time of the backpropagating I1-wave action potential into the dendrites where it initiates the calcium action potential in the dendritic action potential initiation zone (model F in Fig. 1) (Ugawa et al. 2019). While this model is attractive at first sight, it has several shortcomings.: (1) The basis for model F are multiple-electrode patch-clamp recordings in layer V pyramidal cells of rat M1 (Larkum et al. 1999, 2001). These recordings never demonstrated an interval of 1.5 ms or less between the first two or any later action potentials to represent the interval between I1- and I2-waves or later I-waves in epidural spinal cord or SICF recordings, but rather intervals in the order of 5 ms or more (Larkum et al. 1999, 2001; Short et al. 2017). (2) Continuous theta-burst stimulation led to an isolated depression of the I1-wave without effect on late I-waves (Di Lazzaro et al. 2005). Model F cannot explain this finding.

## Clinical and biological relevance of I-waves

SICF measurements reveal that healthy aging is associated with slightly delayed and amplitude-reduced facilitatory peaks (Opie et al. 2018, 2020). This finding should be independent ofm any slowing of conduction along the corticospinal tract because the I-wave intervals of approximately 1.5 ms are independent of conduction velocity of individual corticospinal axons (Edgley et al. 1997). Therefore, prolongation of the intervals between facilitatory MEP peaks likely reflects slowing of impulse conduction along the neural elements responsible for I-waves.

Measurement of SICF may signify abnormal conduction along cortico-cortical fibers in neurological disease. Delayed and/or amplitude-reduced SICF peaks were found in multiple sclerosis, a demyelinating disease of the central nervous system (Ho et al. 1999; Mori et al. 2013).

In contrast, exaggerated SICF peaks were reported in Parkinson’s disease (Ni et al. 2013; Shirota et al. 2019), in particular in those with levodopa-induced dyskinesias (Guerra et al. 2019), and in amyotrophic lateral sclerosis directly related to upper motoneuron signs and disease severity (Van den Bos et al. 2018a), an abnormality that was interpreted as an abnormally hypersynchronized and/or hyperexcitable state of excitatory interneurons in M1.

During voluntary movement, the firing rate of pyramidal tract neurons in monkey M1 rarely exceeds 100 Hz (Evarts 1968; Cheney and Fetz 1980; Evarts et al. 1983). This is much lower than the I-wave frequency of 600 Hz. However, it is unlikely that the I-wave discharge produced by grossly unphysiological stimulation of M1 is merely an artifact without biological relevance. Amassian proposed that the precision of the I-waves serves a timing function (Amassian et al. 1987). Most likely, at least a few inputs need to summate at a corticospinal neuron to produce an I-wave discharge (Creutzfeldt et al. 1966). Thus, corticospinal cells may function like coincidence detectors for inputs arriving through different cortico-cortical and thalamo-cortical projection fibers. This could be tested by triple-coil experiments, with conditioning stimulation over two areas projecting to M1, and test stimulation over M1. One first such study demonstrated a facilitatory interaction of conditioning stimulation over the ventral premotor cortex in combination with conditioning stimulation over the posterior parietal cortex (Shields et al. 2016), but this experiment was not done at the resolution of specific I-wave intervals, and the readout was MEP amplitude rather than SICF.

Moreover, it was demonstrated that distinct I-wave circuits come into play for different forms of hand motor action, e.g., precision vs. power grip (Federico and Perez 2017; Jo and Perez 2019), different forms of motor learning, e.g., model-free learning of a repetitive thumb acceleration task vs. model-based learning of a visuomotor gain adaptation task (Hamada et al. 2014), or different synaptic input into M1, e.g., somatosensory input evoked by peripheral nerve electrical stimulation mediated through vs. bypassing the cerebellum (Hamada et al. 2012, 2014). In all of those studies, the involvement of distinct I-wave circuits was inferred from differential behavior of MEP amplitude and/or SICF when comparing TMS test pulses over M1 that induce current in posterior–anterior vs. anterior–posterior direction, known to result in activation of different sets of I-waves (see above, (Day et al. 1989; Sakai et al. 1997; Di Lazzaro et al. 2001; Delvendahl et al. 2014)).

## Conclusions

What have we learned in the last 20 years, since the original review on the nature of I-waves (Ziemann and Rothwell 2000)? The exact nature of the generation of I-waves, almost 70 years after their first description, is still unclear. But the available evidence predominantly points to different chains of excitatory interneurons that mediate different I-waves. This has been most clearly demonstrated by their differential expression with TMS-induced currents of opposite direction in the motor cortex, and by their selective modulation by a variety of interventions such as conditioning TMS pulses, or repetitive TMS protocols. Pharmacological experiments added important information that the neurotransmitter of the I-wave mediating excitatory interneurons is glutamate, while GABAAergic inhibitory interneurons suppress I-waves. Together, these findings point to circuitry as indicated in model D of Fig. 1 as the most likely neural basis of I-waves. The currently available evidence does not directly support or even speak against alternative I-wave models, such as a high-frequency membrane oscillator (model E in Fig. 1), or apical dendritic backpropagation (model F in Fig. 1). Further progress in our understanding of I-waves will likely come from novel experimental approaches that allow recordings of single corticospinal cell responses to TMS (Mueller et al. 2014; Li et al. 2017).
